# Multiplexed ultrasound beam summation for side lobe reduction

**DOI:** 10.1038/s41598-019-50317-7

**Published:** 2019-09-27

**Authors:** Asaf Ilovitsh, Tali Ilovitsh, Katherine W. Ferrara

**Affiliations:** 1 Department of Radiology, Stanford University, Palo Alto, CA USA; 2 Department of Biomedical Engineering, University of California, Davis, California USA; 3 Department of Biomedical Engineering, Faculty of Engineering, Tel Aviv University, Tel Aviv, Israel

**Keywords:** Biomedical engineering, Ultrasonography, Imaging techniques

## Abstract

Two-way focusing, which relies on sweeping a focused beam across a field of view, is the conventional method for performing high-quality ultrasound imaging. Side lobes resulting from diffraction reduce the image contrast, thus degrade the image quality. In this paper, we present a new method for beam shaping the transmitted ultrasound waveform in order to reduce side lobes and improve image quality. The beam shaping is achieved by transmitting two different waveforms that are interlaced between the odd and even elements. One waveform generates a standard diffraction-limited single focus, and the second waveform generates two foci at the same focal depth as the single focus. The distance between the two foci is selected such that they will destructively interfere with the first order side lobes of the single focus, effectively eliminating these side lobes. A 7.5 dB side lobe reduction was measured experimentally at a depth of 60 mm, using a phased array transducer with a center frequency of 3 MHz. This real-time method utilizes standard receive beamforming, identical to traditional two-way focusing, and does not require post-processing. The method can be implemented with conventional ultrasound systems, without the need for additional components. The proposed method is described analytically, optimized via numerical simulation, and validated by experiments using wire targets, tissue-mimicking phantoms, and *in vivo* imaging of the rat bladder.

## Introduction

Ultrasound imaging is a real time, cost effective, noninvasive and deep penetrating imaging modality that is widely used in medical diagnostics^1,2^. Two-way focusing is one of the most standard ultrasound imaging methods where the transmitted beam is focused to a specific depth^3^, then steered in order to cover a region of interest within the sample^4^. On receive, the standard technique used for image reconstruction is the delay and sum beamforming algorithm^5^. Two-way focusing results in high lateral resolution, high SNR, compatibility with commercial systems and direct control over the field-of-view of the pulse sequence^6^.

As a result of the diffraction limit, the focal shape of a point source is determined by the point spread function (PSF) of the ultrasound system^4^. For a rectangular aperture, the focal spot is approximated by a sinc function composed of a main lobe and side lobes^7^. The resulting ultrasound image is then determined by a convolution between the imaged object and the PSF. Therefore, the presence of side lobes in the PSF degrades the image contrast. In addition, a strong reflector located in the first side lobe can be misinterpreted as a weak reflector on-axis^4^.

In this paper, we propose a method to reduce side lobes based on acoustical beam shaping. Recently, ultrasound beam shaping was used to enhance the performance of two-way focusing through acoustical structured illumination for super resolution imaging^8^, imaging beyond ultrasonically-impenetrable objects^9^, and axial multifoci imaging in a single transmission^10^. In this paper, we propose a beam shaping method for enhancing the contrast of the image, using an idea that is inspired from optical imaging. In optics, stimulated emission depletion microscopy (STED)^11^ is aimed to enhance lateral resolution (rather than reduce the side lobes as proposed here). In STED, two laser beams are transmitted subsequently. The first is an excitation beam that excites the fluorescent molecules in a tagged sample, with a standard diffraction limited PSF. The second beam is a toroid-shaped beam which is used to photobleach the fluorescent molecules in the toroid, leaving only the molecules in the center. Thus the remaining fluorescence stems from a region that is narrower than the diffraction-limited excitation focus^12,13^. Our method utilizes a similar concept, where the side lobe reduction is achieved as a result of the interaction between two waveforms; a single focus and a toroidal waveform. Since standard ultrasound is planar, the planar cross section of a toroid results in two foci. The two waveforms are interlaced within a single transducer such that the first waveform is transmitted by the odd elements, and the second waveform is transmitted by the even elements. The two foci locations are designed to overlap with the first order side lobes of the single focus such that they destructively interfere and thus, an ultrasound side lobe reduction (USLR) is achieved. The USLR method utilizes single-cycle excitation for all elements, and only requires the modification of the transmit delay and apodization maps. This can be later incorporated in a standard B-mode imaging sequence, which makes the method easy to implement in commercial ultrasound systems.

The most common method for side lobe reduction via manipulation of the transmitted field is applying a weighting amplitude (i.e. apodization) across the aperture using various functions including Hamming, Hanning, and Blackman^4,14^. However, the side lobe reduction is gained at the expense of increased width of the main lobe, thus the lateral resolution is degraded. Another approach uses broadband beams such as the ultrasound needle pulse^15,16^. This method theoretically yields a PSF with reduced side lobes. However, this approach requires sending different signals to each element of the transducer and thus cannot be implemented directly in conventional ultrasound systems which use the same signal for all elements and only control the delay and apodization, limiting the current applicability of these methods in the clinic.

Another category of methods achieves side lobe reduction by using innovative beamforming algorithms on reception without modifying the transmitted pulse^17–21^. Although useful, these methods achieve higher performance at the cost of an increased computational complexity, and often cannot be implemented in real-time. These methods can be applied in the future to the proposed method in order to further enhance its performance.

The paper is organized as follows; the design of the transmitted signal is presented first. Then, the method is optimized via numerical simulation. Finally, it is validated through real time experiments in wire targets, a tissue-mimicking phantom, and an *in vivo* biological sample.

## Design of the Transmitted Signal

**Figure 1:**
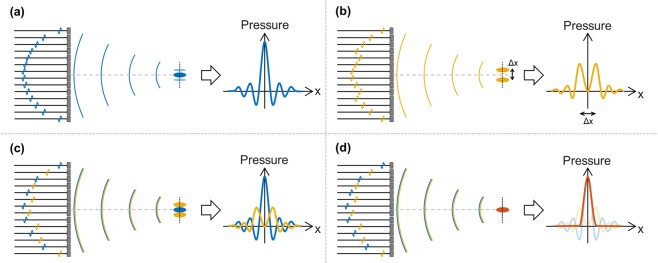
Schematic illustration of the proposed USLR method. (**a**) The first waveform generates a single focus at a specific focal depth. (**b**) The second waveform generates two foci at the same focal depth, with a Δx lateral distance between the foci. (**c**) Interlacing the two waveforms, and adjusting the amplitude such that the main lobes of the two foci will cancel the side lobes of the single focus. (**d**) The resulting PSF with reduced side lobes.

The proposed implementation of this USLR method is based on interlacing two waveforms. The first waveform is a standard single focal transmission (Fig. 1a). Each element transmits a single-cycle pulse at a specific time point, and the delay in transmission between different elements is set such that all pulses will interfere constructively. The second waveform (Fig. 1b) generates two foci with a lateral distance of Δx between them. The distance is chosen such that the foci overlap with the first order side lobes of the single focal transmission. The two foci waveform was designed using the pseudo-inverse method^22^. The two waveforms are then interlaced together such that odd elements transmit the single focus and the even elements transmit the two foci waveform (Fig. 1c). The magnitude of the two foci waveform is adjusted to cancel the side lobes of the single focus transmission, and result in a PSF with reduced side lobes (Fig. 1d).

## Simulation and Optimization

The transmitted waveform (WF) using the USLR method can be expressed by the following equation:

1 $$WF=W{F}_{singlefocus}[odd\,elements]+c\cdot W{F}_{twofoci}(\Delta x)[even\,elements]$$

where WF_single focus_ and WF_two foci_ are the waveforms for single focus and two foci respectively, c is the amplitude adjustment coefficient for WF_two foci_, and Δx is the lateral distance between the two foci.

**Figure 2:**
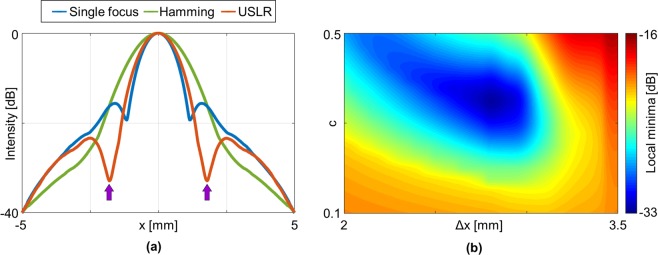
Simulation and optimization results. (**a**) Simulation results for standard single focus at 60 mm (blue), apodization using a Hamming window (green), and the proposed USLR method (red) with c = 0.35 and Δx = 2.85. (**b**) Optimization process for finding the optimal parameters by minimizing the first order side lobes, yielding a local minima at c = 0.35 and Δx = 2.85.

Acoustic pressure fields were simulated using the following parameters (selected to correspond with the experimental setting): a 128-element array with a 0.22 mm pitch, a center frequency of 3 MHz and a focal depth of 60 mm, corresponding to the elevation focus of this array. First, a standard single focus at 60 mm was simulated (blue curve in Fig. 2a). The two first order side lobes are located at ±1.6 mm. Next, a Hamming window was simulated (green curve in Fig. 2a), where the Hamming window was chosen as it is considered the function that yields the best compromise between the contrast of images and the lateral resolution^23^. While the Hamming window reduces the side lobes, it also increases the main lobe width by 49%, from 1.03 mm to 1.53 mm (measured at −3 dB). The optimized USLR waveform (red curve in Fig. 2a) reduces the first order side lobes in the blue curve by 17.3 dB (from −15.6 dB to −32.9 dB), generating local minima marked by purple arrows in Fig. 2a. The optimized waveform parameters were selected using an optimization process with the variables c and Δx, to yield the maximal reduction of the first order side lobes in standard two-way focusing. The local minima as a function of c and Δx is smallest at c = 0.35 and Δx = 2.85 mm, with a value of −32.9 dB (Fig. 2b). Using these parameters, the main lobe width increases by 9% to 1.12 mm.

Grating lobes are an artifact generated as a result of undersampling of the spatial frequencies in the transmitted and received ultrasound beams, and depend on the transmitted wavelength and the element size and spacing^24^. In phased array transducers with a pitch below one-half wavelength, grating lobes are not generated (Supplementary Fig. S1a). Since the USLR method interlaces two waveforms on transmit, the effective pitch of the array on transmission is doubled and grating lobes are generated. The resulting beam profile is similar to that resulting from generation of a single focus using only the odd elements while setting the even elements to zero (Supplementary Fig. S1b, left). The grating lobe intensity on transmission is similar for the conditions in Supplementary Fig. S1b,c, and is suppressed by 30 dB compared to the main lobe. Although the USLR method interlaces the two waveforms on transmission, the entire aperture is used for receive. Therefore, with an effective pitch below one-half lambda on receive, grating lobes are absent^25^. The two-way focused profile is the product of the transmission and reception profile, and as a result the grating lobes are suppressed by 80 dB compared to the main lobe (Supplementary Fig. S1c, right).

## Experimental Results

### Hydrophone measurements

**Figure 3:**
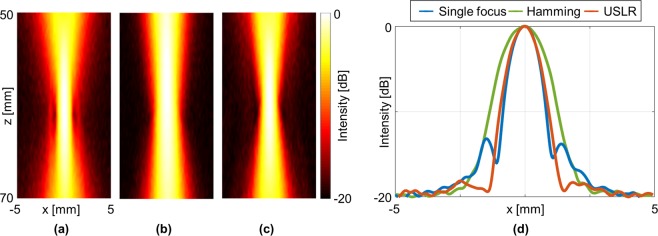
Hydrophone measurements of the emitted acoustic pressure fields. Axes are common to (**a**–**c**). PSF of (**a**) Single focus at 60 mm, (**b**) Using a Hamming window, and (**c**) Using the USLR method. (**d**) Normalized lateral profiles of the PSFs at 60 mm.

Validation of the simulation was performed by measuring the emitted pressure field using a needle hydrophone (Fig. 3). For standard single focus at 60 mm, the average side lobe intensity is −13.5 dB compared to the main lobe (Fig. 3a), where average refers to the average side lobe level of the left and right side lobes for a single main lobe. The side lobes are reduced while using a Hamming window, at the expense of an increase of the main lobe width by 55% from 0.99 mm to 1.52 mm (Fig. 3b). Using the USLR method, the side lobes are reduced by 5.8 dB to an average value of −19.3 dB, with a main lobe width increase of 7% to 1.06 mm (Fig. 3c). The normalized lateral profiles of the three PSFs at 60 mm are presented in Fig. 3d.

### Wire target experiments

**Figure 4:**
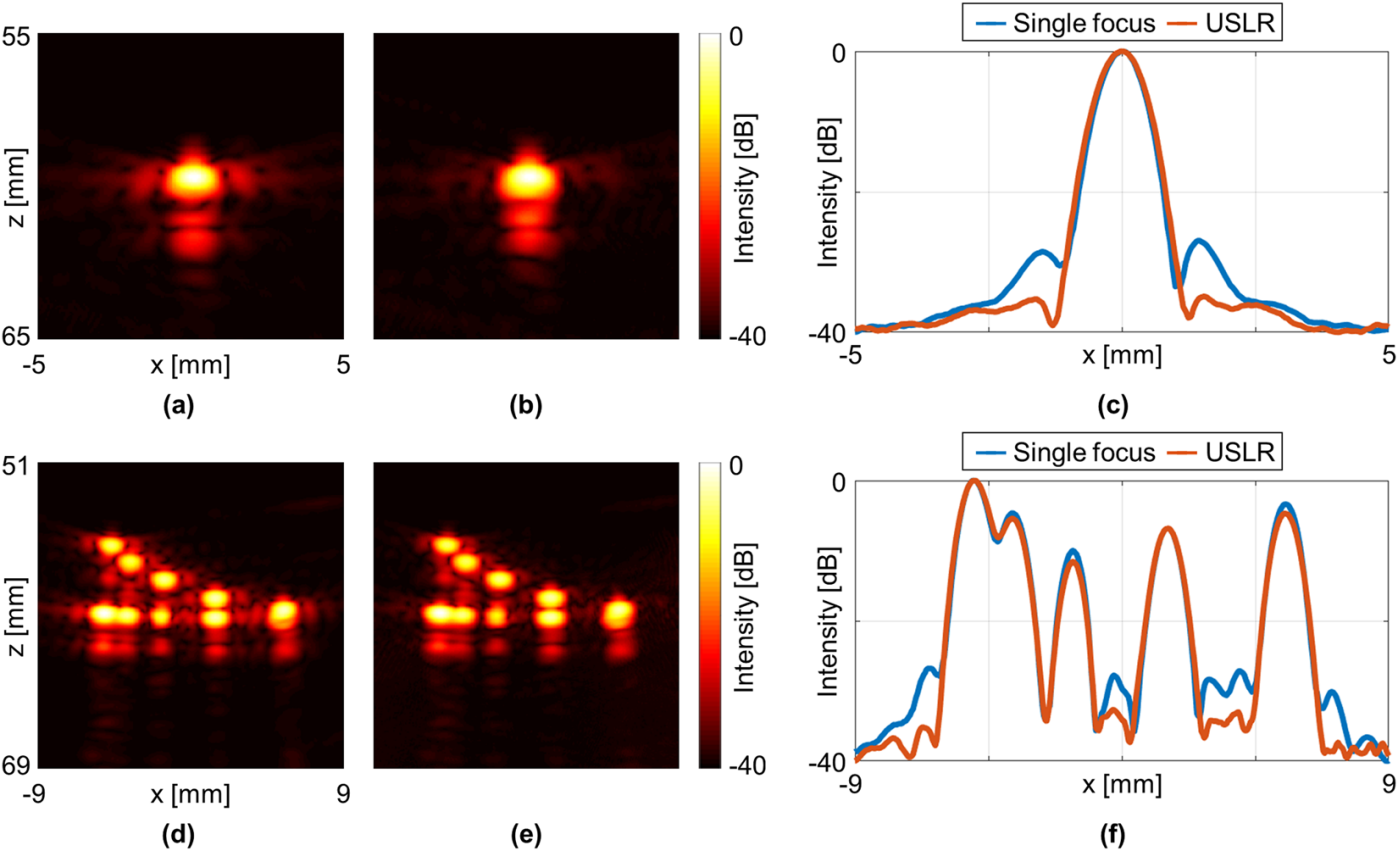
Experimental results; wire targets in water. (**a**) Single wire using a single focus at 60 mm and rectangular apodization window. (**b**) Single wire using the USLR method. (**c**) Normalized lateral profiles of the single wire target. Multiple wire targets using (**d**) Single focus at 60 mm and rectangular apodization (**e**) USLR method. (**f**) Normalized lateral profiles of the wire targets.

To validate the USLR method and effectively demonstrate the side lobe reduction, a single 50-µm nylon wire was positioned in a water tank at a depth of 60 mm. Transmission of a standard single focus at 60 mm using a rectangular apodization window (Fig. 4a) resulted in side lobes with an average intensity of −27.7 dB compared with −35.2 dB for the USLR method (Fig. 4b), a reduction of 7.5 dB. The normalized lateral profiles of the two PSFs are presented in Fig. 4c where the left side lobe is reduced from −28.5 dB to −35.3 dB, and the right side lobe from −26.9 dB to −34.9 dB. The width of the USLR PSF increased by 5%, from 0.90 mm to 0.95 mm (measured at −6 dB), and the main lobe peak intensity decreased by 6.4 dB. This reduction in peak intensity is expected, as one half of the elements transmit the single focus.

A phantom containing 11 wires (see methods) was then imaged using a single focus at 60 mm (Fig. 4d), and the USLR method with the optimized parameters as above (Fig. 4e). The resolution obtained with the two methods is comparable, while the side lobe reduction obtained with the USLR method is evident for all wires as confirmed by the lateral intensity of the wires at a depth of 60 mm (Fig. 4f).

### Contrast measurements

**Figure 5:**
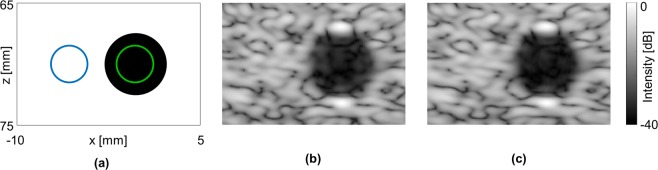
Experimental results; cyst in a tissue-mimicking phantom. Axes are common to subfigures (**a–c**). (**a**) Phantom cyst illustration, where the green and blue circles mark the cysts and speckle positions, respectively. (**b**) Single focus at 70 mm. (**c**) USLR at 70 mm, using c = 0.37 and Δx = 3.05 mm. Images are normalized to a peak intensity at 0 dB.

The CIRS 040GSE commercial tissue-mimicking phantom was then used to measure the contrast and contrast to noise ratio (CNR) of a cyst at depth of 70 mm. An illustration of the cyst phantom is presented in Fig. 5a, where the green and blue circles mark the cyst and speckle positions that were used for the calculations, respectively.

The contrast is defined as the difference in brightness between the cyst and the surrounding region, and it is defined by:

2 $$Contrast[dB]=20\,{\log }_{10}(\frac{{\mu }_{i}}{{\mu }_{o}})$$

where µ_i_ is the mean of a region inside the cyst, and µ_o_ is the mean of the background speckle region outside the cyst.

The CNR provides a metric for cyst detection compared to the surrounding background. It is defined by:

3 $$CNR[dB]=20\,{\log }_{10}(\frac{{\mu }_{o}-{\mu }_{i}}{\sqrt{{{\sigma }_{o}}^{2}+{{\sigma }_{i}}^{2}}})$$

where *σ*_*i*_ is the standard deviation of a region inside the cyst, and *σ*_*o*_ is the standard deviation of the background speckle region outside the cyst^26,27^.

The cyst was imaged based on transmission of a single focus at 70 mm (Fig. 5b) and with the USLR method (Fig. 5c). Here, the USLR parameters were c = 0.37 and Δx = 3.05 mm, which were the optimized parameters for this depth. The contrast increased by 2.9 dB, from −30.4 dB using the single transmission focus to −33.3 dB using the USLR. The CNR increased by 0.6 dB, from 10.9 dB using single focus to 11.5 dB using the USLR. Thus, both the contrast and the CNR are improved using the USLR method.

### *In vivo* experiment

**Figure 6:**
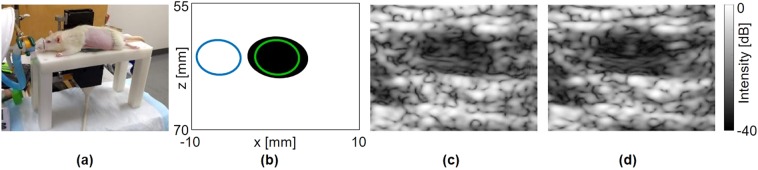
Experimental results; *in-vivo* rat bladder. Axes are common to subfigures (**b–d**). (**a**) Image of the *in vivo* experimental setup. (**b**) *In vivo* bladder illustration, where the green and blue circles mark the bladder and speckle positions, respectively. Ultrasound image of the bladder using (**c**) Single focus at 60 mm; (**d**) USLR at 60 mm.

In the final experiment, the USLR method was applied to image the rat bladder *in vivo*, where side lobe artifacts are expected to be more pronounced^28^. The animal was placed on an in-house designed bed and the P6-3 transducer was located beneath an agarose window facing upwards (Fig. 6a). An illustration of the bladder is presented in Fig. 6b, where the green and blue circles mark the cyst and speckle locations that were used for the calculations, respectively. The bladder was imaged using a single focus at 60 mm (Fig. 6c) with the USLR method (Fig. 6d), and the contrast and CNR were calculated. Using the USLR method, the contrast increased by 2.8 dB (from −10.9 dB to −13.7 dB), and the CNR increased by 1.1 dB (from −0.3 dB to 0.8 dB).

## Discussion and Conclusions

The USLR method is a real-time method where two waveforms are interlaced to reduce side lobes and enhance the image contrast. Side lobes are an unwanted ultrasound artifact originating from the finite-sized ultrasound transducer aperture^7^. These artifacts increase noise and reduce image contrast. Side lobe artifacts are pronounced in high dynamic range imaging applications, and especially in abdominal imaging near curved, highly reflective surfaces such as the diaphragm and the gas-filled bowel, near large cystic masses or on an anechoic background such as the urinary bladder or gallbladder^28^. In the urinary bladder, side lobe artifacts are also increased by the reflective interface between urine and bladder wall and due to adjacent gas in the colon^29^. Both in the urinary bladder and the gallbladder side lobes contribute to the formation of apparent echogenic material on the distal boundary of the urinary bladder, resulting in an artifact known as pseudo-sludge^30^. Therefore, the method is anticipated to be useful in these applications. The method can be incorporated in a regular imaging protocol, by changing the transmitted apodization and delay according to the optimized values, which are calculated only once (prior to imaging). While apodization window functions (e.g. Hamming) can also reduce side lobes, the main lobe width increases and the lateral resolution is degraded. Alternatively, the USLR method achieves side lobe reduction without significantly increasing the main lobe (less than 10%).

The USLR method performance was compared experimentally to two-way focusing, which is the dominant beam formation method in commercial ultrasound systems. The USLR method was initially validated using a wire target, and an average side lobe reduction of 7.5 dB was achieved. Next, the impact on resolution and side lobes was further evaluated using multiple lateral and axial wire targets. The proposed method is intended to reduce the side lobes at a specific focal depth, similar to two-way focusing that generates a single focus at a specific depth. Therefore, side lobes are reduced for multiple lateral targets at the focal depth (Fig. 4f). While outside the focal depth, the side lobe are not reduced, the proposed method does not compromise the spatial resolution for the axial targets. The method was then used to image a cyst in a tissue-mimicking phantom. The contrast and the CNR were increased by 2.9 dB and 0.6 dB, respectively. This improvement is attributed to the side lobe reduction, despite the fact that the generated focal intensity decreases by 6.4 dB compared to the standard single focus method. The intensity reduction stems from the fact that only half of the elements contribute to the generation of the focal volume. For most clinical imaging applications, the transmitted intensity is limited^31^ to remain below a mechanical index of 1.9 (defined as the peak negative pressure divided by the square root of the center frequency^32^). For imaging scenarios which are limited by the mechanical index (such as abdominal imaging of obese patients), the intensity transmitted by each element with the USLR method can be increased by 6 dB as compared to conventional imaging while maintaining the same mechanical index. As a result, the method’s performance is expected to be further improved. Finally, the method was tested for *in vivo* imaging of the rat bladder, where the contrast and CNR were increased by 2.8 dB and 1.1 dB, respectively.

The proposed method interlaces the even and odd elements, and effectively doubles the pitch on transmission for each of the two waveforms. Since a phased array transducer with a pitch close to half a wavelength was used here, the effective pitch for each of the transmitted waveforms was close to one wavelength and was thus similar to a linear array transducer. For this case, on transmission, grating lobes are generated at large deflection angles (> ±45°)^24^. In the USLR method, all elements are used on reception with the original half-lambda element spacing, and as a result grating lobes are not generated on receive. The two-way profile is the product of the transmission and reception profiles and therefore the grating lobes are suppressed^25^.

The elevation focus of the transducer used here was 60 mm, and therefore it was our natural choice to choose this imaging depth. However, the method is applicable to any practical focal depth. For the transducer used here and similarly the analysis carried in Fig. 2g in^10^, the directivity of the outer elements was not sufficient for a focal depth less than 10 mm, hence focusing to shallower depth cannot be accomplished using the full aperture for this given transducer. Therefore, focusing to a shallower depth requires a reduced aperture. If side lobe reduction over a larger depth is desired, the method can be incorporated into a successive focusing imaging scheme where the transmission focal depth is updated successively with several transmissions^33^.

While here the USLR method was used for imaging, and therefore used a single cycle excitation in order to maximize the axial resolution, the method can also be considered for applications that require longer pulses (such as pulsed wave Doppler or color flow). However, the method is designed to eliminate the first-order sidelobe. The transmission of longer pulses will generate higher order sidelobes which the method is not designed to eliminate.

Here, the USLR method was applied with a relatively low ultrasound frequency of 3 MHz, which dictated the lateral resolution; however, applying the technique with a substantially higher frequency will improve the resolution in proportion to the increased frequency. Current ultrasound arrays are feasible up to ~40 MHz^34–36^. Implementation of the USLR method in 3D ultrasound imaging is also feasible. Two-dimensional transducer arrays are under development for radiology applications^37^. Currently, many of these transducers are limited to relatively low frequencies which limits their performance. Applying the USLR method with these transducers is expected to enhance the resulting 3D image quality.

In conclusion, we have presented a new optically-inspired method for improving the contrast of an ultrasound image by lowering the side lobes, without reducing the resolution or frame rate.

## Methods

### USLR method

The USLR method requires the following steps: (1) determine the depth to be imaged, (2) calculate the transducer phase and apodization maps for the single and two foci transmissions using the pseudo-inverse method^22^, (3) interlace the two waveforms and simulate the emitted field, (4) optimize the variables c and Δx in order to achieve the maximal reduction of the first order side lobes and (5) incorporate the resulting phase and apodization maps in the Verasonics imaging routine.

### Numerical simulations

Simulation and post processing of the images were implemented in MATLAB (version 2018a, MathWorks, Natick, MA, USA). The acoustic pressure fields were simulated using Field II software^38,39^. Both programs run on a Dell OptiPlex 7040 PC with a Windows 10 Enterprise 64-bit operating system, Intel® Core™ i7-6700 processor, 3.40 GHz, 16 GB RAM. Using this simple PC, and using our code (which was not optimized for enhanced run time), simulation of a single slice required ~1 second. A single 2D numerical simulation required ~3 minutes, while an optimization routine such as the one presented in Fig. 2b required ~5 minutes to run.

### Ultrasound imaging

Imaging was performed using the Verasonics ultrasound system (Vantage 256, Verasonics Inc., Kirkland, WA, USA), with a phased array sector transducer P6-3 (Philips, Bothell, WA, USA) operating at a center frequency of 3 MHz, with an elevation focus at 60 mm. This array has 128 elements with a 0.22 mm pitch and therefore a total aperture of D = 28.2 mm. The excitation for each transmitted pulse was a single cycle waveform. Imaging was performed with 256 angles over an 18° field of view, resulting in an angular precision of 0.07°. This high angular precision was selected for fine sampling of the PSF. Beamforming was implemented in real-time using the Verasonics software, with its build-in scripts in Matlab.

### Hydrophone measurements

The acoustic pressure field was measured in a degassed water tank using a wide-band needle hydrophone (HNP-0400, Onda, Sunnyvale, CA, USA) with an active aperture of 0.4 mm. The hydrophone probe was mounted on a three-dimensional positioning system (Newport motion controller ESP 300, Newport 443 series. The pressure signals received by the hydrophone were first displayed on a digital oscilloscope (DPO4034, Tektronix, OR, USA), and further recorded and converted via post processing in Matlab to a normalized pressure map.

### Resolution wire target phantom

A resolution wire target was designed in-house, and contained eleven 50 µm nylon wires with an axial separation of 4, 3, 2, 1, and 0.5 mm, and a lateral separation of 4, 3, 2, 1, and 0.5 mm.

### Tissue-mimicking phantom

A tissue-mimicking phantom with attenuation of 0.5 dB/cm/MHz (CIRS 040GSE, Virginia, USA), consisting of a Zerdine^®^ hydrogel polymer^40^, was used for testing the contrast and CNR.

### In vivo

All animal-related work performed by our laboratory was in accordance with the Guide for the Care and Use of Laboratory Animals of the National Institutes of Health (NIH) and all animal experiments were performed under a protocol approved by the Institutional Animal Care and Use Committee (IACUC) of the University of California, Davis. All experiments were performed in accordance with relevant guidelines and regulations.

The *in vivo* experiment was performed on a Sprague-Dawley rat (Charles River, Wilmington, MA). Prior to the experiment, fur around the imaging area was shaved and then further removed using depilatory cream. The animal was placed in a prone position and imaging was performed on the sagittal plane. Ultrasound gel was used as a coupling agent. During imaging, the animal was maintained under anesthesia using 1–2% isoflurane in oxygen (2 L/min) and body temperature was maintained at 37 °C.

### Agarose spacer

1.5% Agarose (Alfa Aesar, MA, USA) was used for fabricating an ultrasound spacer that was used in the *in vivo* experiment. The Agarose powder was mixed with deionized water at ambient temperature and heated until all powder was dissolved. The solution was degassed under vacuum, then poured into a mold and allowed to cool at ambient temperature.

## Supplementary information

Supplementary Information

## Data Availability

The data sets generated during and/or analyzed during the current study are available from the corresponding author on reasonable request.
