# Supplementary material for: Multiplexed ultrasound beam summation for side lobe reduction
Source: Sci Rep. 2019 Sep 27;9:13961. doi: 10.1038/s41598-019-50317-7 (PMC6765004; doi:10.1038/s41598-019-50317-7)
Supplement: Supplementary file 1 — Supplementary Information [file 41598_2019_50317_MOESM1_ESM.pdf]

Supplementary Information

Multiplexed ultrasound beam summation for side lobe reduction

Asaf Ilovitsh[1,2], Tali Ilovitsh[1,2,3], and Katherine W. Ferrara[1,*]

[1] Department of Radiology, Stanford University, Palo Alto, CA, USA

[2] Department of Biomedical Engineering, University of California, Davis, California, USA.

[3] Department of Biomedical Engineering, Faculty of Engineering, Tel Aviv University, Tel Aviv, Israel.

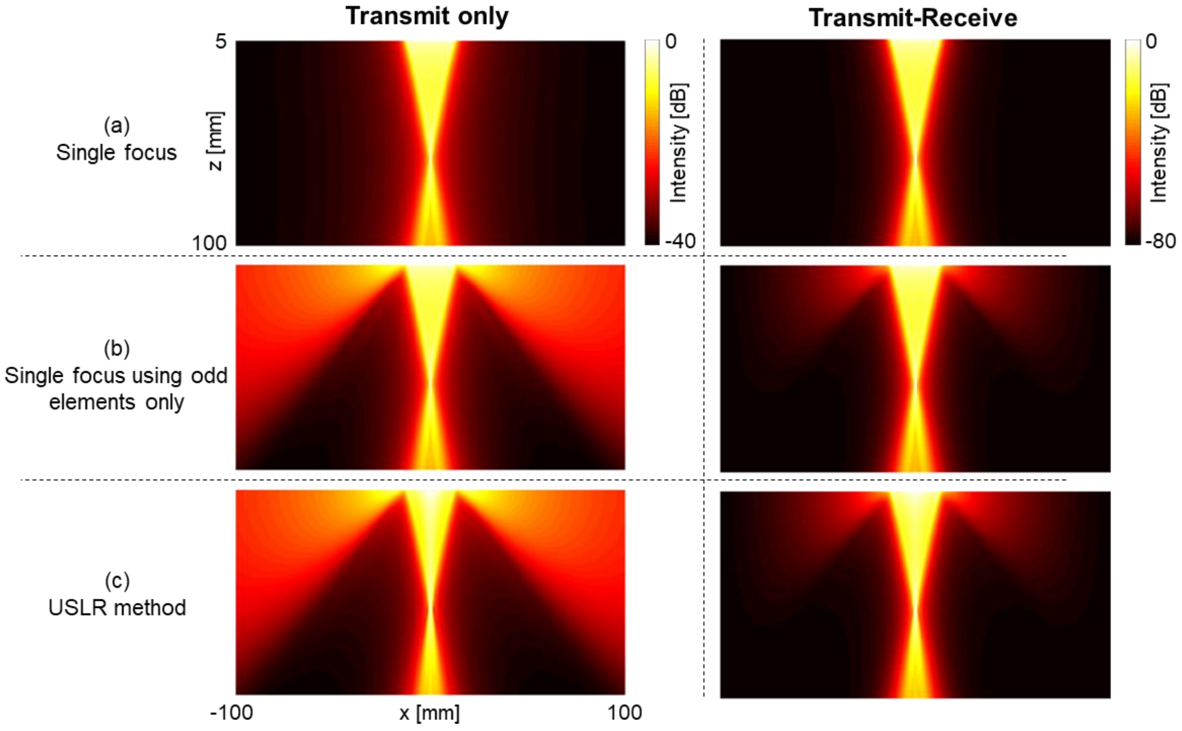

Supplementary Fig. S1. Grating lobes on transmit and transmit-receive beamforming for three transmission methods: (a) Single focus. Full aperture is used both on transmit and on receive. (b) Single focus transmitted using the odd elements (the even elements are set to zero). The entire aperture is used on receive. (c) The USLR method that interlaces two waveforms on transmit and uses the full aperture on receive. The transmit waveforms are presented with a 40-dB dynamic range and the transmit-receive waveforms are presented with an 80-dB dynamic range.
